# Preoperative Immunonutrition and Postoperative Outcomes in Radical Pancreaticoduodenectomy Patients

**DOI:** 10.5152/tjg.2023.22793

**Published:** 2024-01-01

**Authors:** Murat Aba, Ahmet Gökhan Sarıtaş, Burak Yavuz, Kubilay Dalcı, Abdullah Ülkü, Atılgan Tolga Akçam

**Affiliations:** 1 Department of General Surgery, Diyarbakır Selahattin Eyyubi Devlet Hastanesi, Diyarbakır, Turkey; 2 Department of General Surgery, Çukurova University Faculty of Medicine, Adana, Turkey

**Keywords:** Pancreatic neoplasms, enteral nutrition, pancreaticoduodenectomy

## Abstract

**Background/Aims:**

This study aimed to compare the patient groups who received and did not receive immunonutrition in terms of mortality and morbidity in patients who underwent radical pancreaticoduodenectomy.

**Materials and Methods:**

Two groups were formed from 40 patients who underwent radical pancreaticoduodenectomy in our clinic in 2021. The patients in study group were given enteral immunonutrition support for 5 days preoperatively. For this purpose, a standard enteral immunonutrition product containing arginine, omega-3 fatty acids, and RNA (dietary nucleotides) was used. Patients’ data of demographical, laboratory, postoperative complications, and current clinical status were analyzed.

**Result:**

Mortality developed in 5 (25 %) patients in the treatment group and 4 (20 %) patients in the control group in the following months (*P* > .05). The estimated survival rate in the treatment group was 21.8 ± 2.8 months in the treatment group 19.1 ± 1.7 months in the control group (*P* > .05). The length of hospital stay was 12.89 ± 3.3 days in the treatment group, while it was 16.47 ± 6.83 days in the control group (*P* < .05). In the postoperative follow-ups, delayed gastric emptying symptoms developed in 3 patients in the treatment group, while the same complication was observed in 9 patients in the control group (*P* < .05). Surgical site infections occurred in 4 patients in the treatment group and 9 patients in the control group (*P* < .05).

**Conclusion:**

It was observed that preoperative oral immunonutrition before pancreaticoduodenectomy was effective in reducing the risk of delayed gastric emptying after surgery and the length of hospital stay.

**Main Points**

- Pancreaticoduodenectomy is an operation with high morbidity rates, and complications include pancreatic fistula, delayed gastric emptying, and infectious complications.
- This procedure induces systemic inflammatory response characterized by the secretion of proinflammatory cytokines.
- Preoperative immunonutrition support decreases postoperative delayed gastric emptying and length of stay in patients undergoing pancreaticoduodenectomy for periampullary tumors.

## Introduction

Periampullary region cancers are defined as tumors originating from the tissue 2 cm around the papilla. These tumors are duodenal cancers, distal common bile duct cancers, Ampulla of Vater cancers, and pancreatic head cancers, in that order of frequency.^1^ Periampullary tumors, the incidence of which increases with age, are diseases with high mortality and morbidity. It is more common in men than women. Pancreaticoduodenectomy (PD) is the only valid curative treatment of periampullary region tumors.^2^ Pancreaticoduodenectomy is still associated with high morbidity due to factors associated with pancreatic surgery and postoperative infectious complications. The postoperative morbidity rate ranges from 40% to 60%,^3^ while the postoperative mortality rate ranges from 3% to 5%.^4^ Complications after PD include pancreatic fistula, delayed gastric emptying (DGE), and infectious complications (wound infections, pneumonia, urinary tract infections).^5^

Low-calorie diets and bacterial translocation were reported to impair the host response and reduce immunological activity in patients undergoing PD, and alterations in postoperative intestinal motility and loss of mucosal barrier function were also discovered to be contributory factors.^6^ This decline in immune function raises the danger of infectious complications, raises medical expenses, and lengthens hospital stays. The nutritional assessment of all patients during the preoperative phase is one of the most crucial steps of the methods intended to prevent morbidity.^7^ Among the proposed strategies to reduce complications, enteral diets supplemented with arginine, omega-3 fatty acids, and ribonucleic acids have been suggested to improve the inflammatory response and wound healing through the provision of essential nutrients involved in T-lymphocyte activity and other immune functions.^8^

Our study’s objective was to assess, in light of recent medical literature, the impact of preoperative immunonutrition in patients who underwent radical PD with the diagnosis of periampullary tumor.

## Materials and Methods

This study was carried out with the approval of Çukurova University Faculty of Medicine Non-Invasive Clinical Research Ethics Committee (ethics committee decision numbered 2020/101-17). Written informed consent was obtained from all patients in the preoperative period.

Forty patients who underwent PD with the diagnosis of periampullary tumor between 2020 and 2021 in Cukurova University Department of General Surgery were included in the study. 

The patients were randomized and divided into 2 groups: the study group (n = 20) and the control group (n = 20). Groups were formed by matching 1:1 according to the order of consecutive clinical presentations. The patients in the study group were given an enteral immune nutrition (EIN) product containing arginine, omega-3 fatty acids, and RNA (dietary nucleotides) for 5 days in the preoperative period. This dietary supplement, which was administered 3 times a day (237 mL/piece), was followed and recorded in the prospective database. The contents of the immunonutrition product are summarized in Table 1.

We provided a standard dose of enteral nutrition to all patients in the treatment group, which is in line with the recommendations for immunonutrition support in major surgery patients. Patients’ tolerance to enteral nutrition was closely monitored throughout the preoperative period. Any adverse reactions or side effects, such as gastrointestinal discomfort or diarrhea, were recorded, and the immunonutrition regimen was stopped if the patient developed intolerance.

Standard PD was performed on all patients by the same surgical team. Patients with tumors that were considered unresectable intraoperatively, patients with chronic diseases (chronic liver disease, renal failure, immunosuppression), and patients with malnutrition (BMI <20 kg/m^2^) were excluded from the study.

The following clinical variables for each patient were analyzed and recorded in the prospective database: age, gender, body mass index (BMI), habits (smoking and alcohol), ASA score, NRS score, concomitant comorbid diseases (DM, HT, and CAD), complaint at the time of application (jaundice and weight loss), blood group, CA 19-9 and carcinoembryonic antigen (CEA) values at the time of diagnosis, survival time, preoperative 5th day and postoperative 5th day laboratory values (white blood cells, hematocrit, platelet count, aspartate transaminase, alanine transaminase, alkaline phosphatase, gamma-glutamyl transferase, blood urea nitrogen, creatine, amylase, total bilirubin, direct bilirubin, C reactive protein, albumin, transferrin, total protein, IgA, INR, Cd-4, and Cd-8), perioperative and postoperative complications, length of hospital stay, and histopathological features of the tumor.

### Surgical Technique

All surgeries were performed by the same surgical team. All patients underwent standard Whipple procedure with regional lymphadenectomy and distal gastrectomy. Pancreatico-jejunal, gastro-jejunal, and hepatico-jejunal reconstructions were standardized and the same anastomosis technique was used in all patients. In the biliary and pancreatic anastomoses, no stent was inserted.

### Definitions

Survival was calculated as the time from the clinical diagnosis to the last control date in surviving patients, and as the time from diagnosis to death for any reason in patients who developed mortality. Perioperative mortality was defined as death occurring before discharge or 30 days postoperatively. Overall mortality was defined as death that occurred during the follow-up period. Morbidity was defined as all complications that developed between surgery and discharge or re-admission.

Delayed gastric emptying was defined as continued need for nasogastric drainage for more than 7 days after PD and reintroduction of parenteral nutritional support. Pancreatic fistula was defined as the coming of drainage fluid with high amylase content (3 times the serum amylase level) from the surgical drain for more than 3 days postoperatively or percutaneous drainage of the intra-abdominal fistula collection. Bile flow from intra-abdominal drains was defined as biliary fistula. Infection requiring removal of sutures from the wound was defined as wound infection.

### Postoperative Management

Total parenteral nutrition (TPN) support was given to all patients on the first postoperative day. The energy requirement was met as 25-30 kcal/kg/day. The fluid deficit of the patients was calculated by following up daily intake and output. TPN and fluid replacements were given via a central catheter. All patients received short-term antibiotic prophylaxis. Low-molecular-weight heparin prophylaxis was started in accordance with the protocol in patients whose hemorrhage risk was excluded.

### Statistical Analysis

Statistical analyzes in our study were performed using Excel and Statistical Package for Social Sciences version 26.0 (IBM Corp.; Armonk, NY, USA). Parametric quantitative variables were expressed as mean ± SD. Categorical variables were expressed as percentages. Survival times of the patients were calculated using the Kaplan–Meier method. A comparison of prognostic factors was done using the Log-rank test, and independent prognostic factors affecting survival were determined by Cox hazard regression test. A *P*-value of <.05 was considered significant.

## Results

The general demographic characteristics of the patients are summarized in Table 2. There was no significant difference between the 2 groups in terms of age, gender, BMI, ASA score, comorbidities, blood type, smoking, and alcohol use. There was no significant difference between the 2 groups in terms of preoperative NRS scores and nutritional status, which is also demonstrated in Table 2.

**Figure 1.**
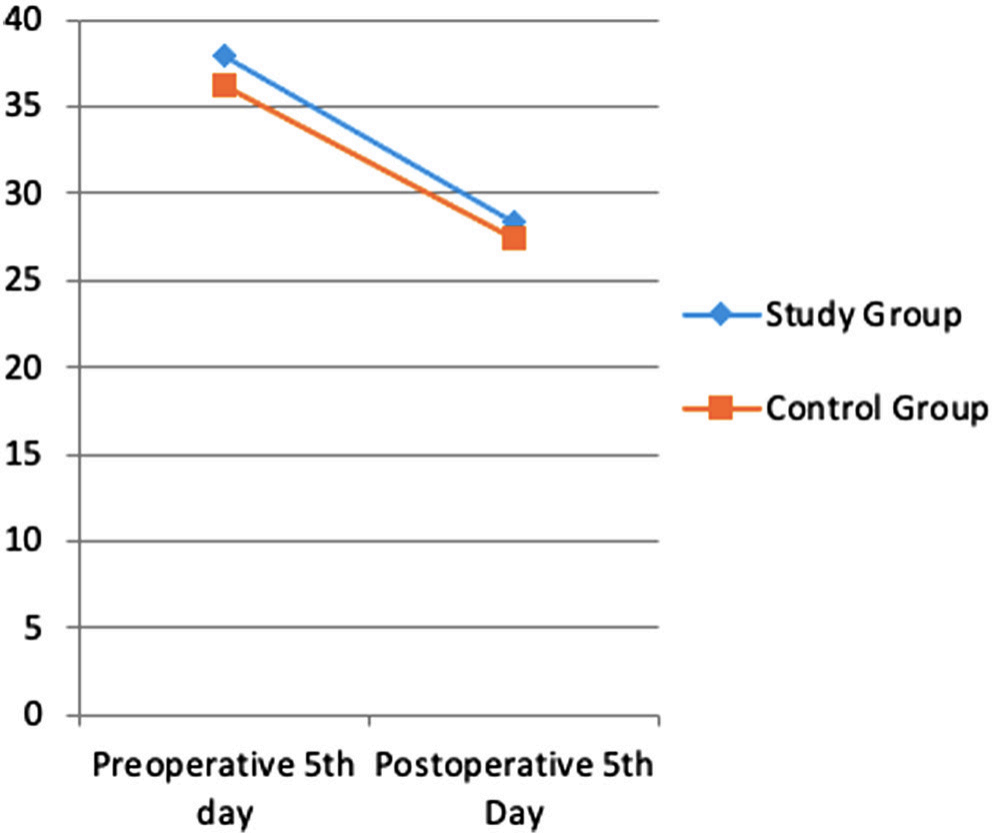
Difference in albumin values between pre-op and post-op.

**Figure 2.**
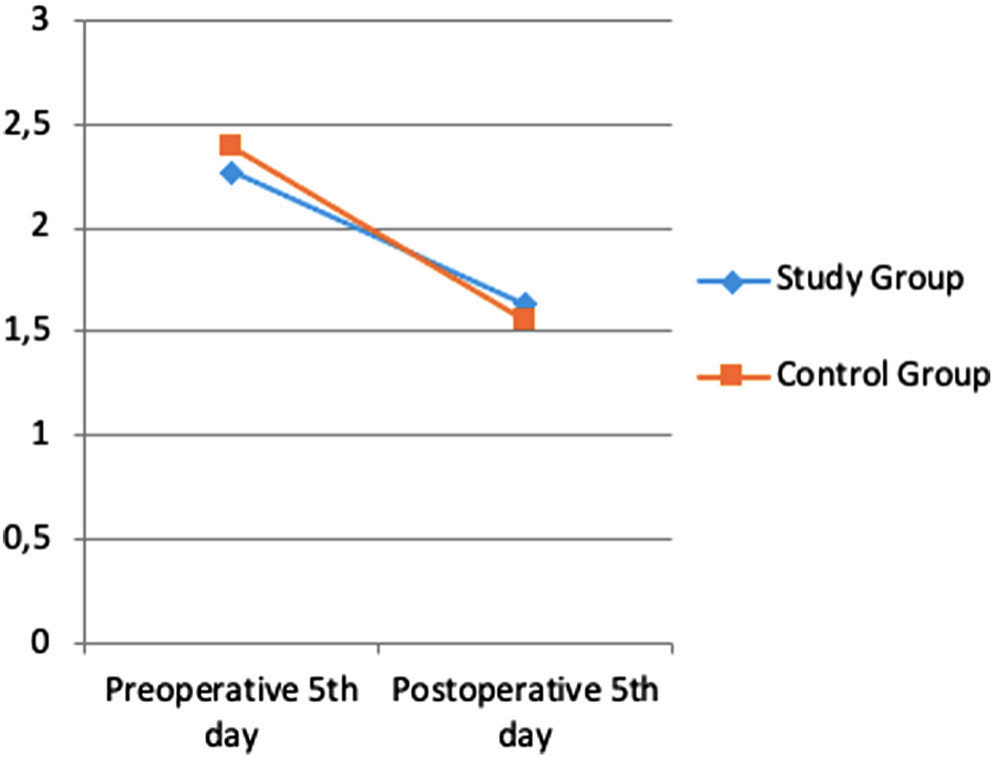
Difference in transferrin values between pre-op and post-op.

**Figure 3.**
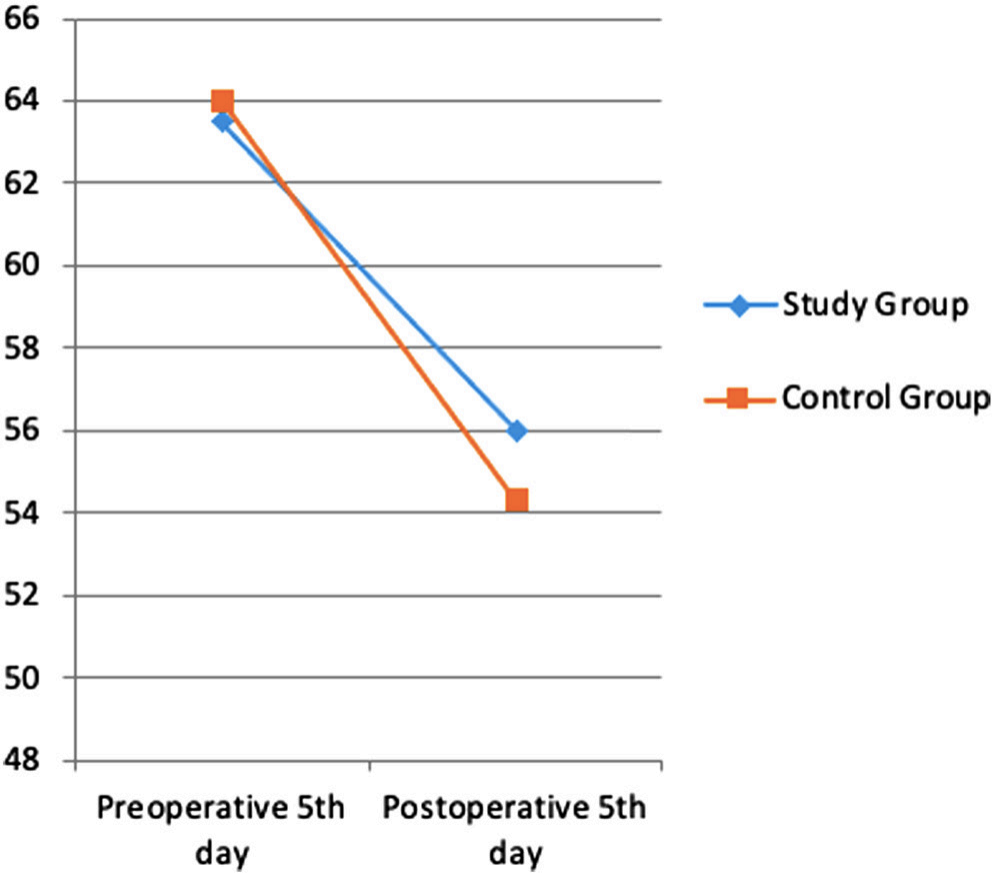
Difference in total protein values between pre-op and post-op.

Albumin, transferrin and total protein levels were examined as indicators of preoperative and postoperative nutritional status. Considering the pre- and postoperative periods in albumin levels, the rates of decrease in both groups were similar (Figure 1). A more limited decrease was observed in transferrin (Figure 2) and total protein (Figure 3) levels in the study group. However, these changes were not found to be statistically significant.

**Figure 4.**
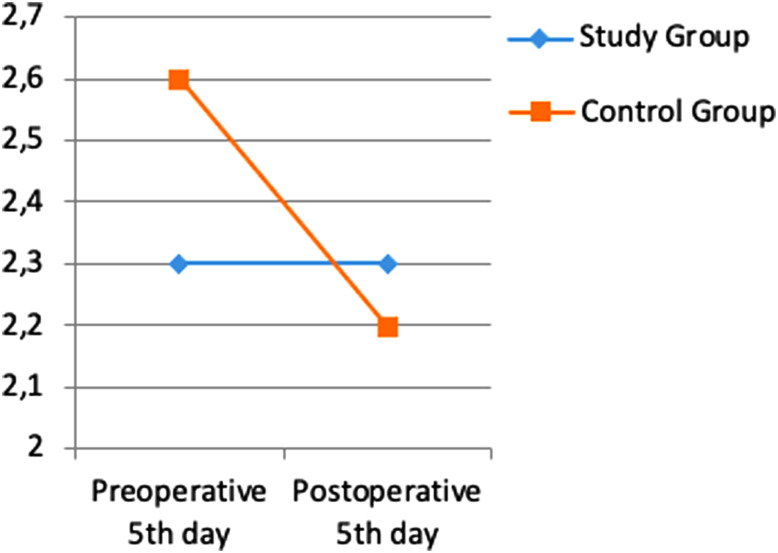
Difference in IgA values between pre-op and post-op.

When Ig A levels, which is one of the most important immune markers in terms of mucosal defense functions, are compared. It was determined that the mean level was preserved in the study group in the pre- and postoperative period, but decreased in the control group. This decrease was not statistically significant (*P* = .253/*P* = .718) (Figure 4).

**Figure 5.**
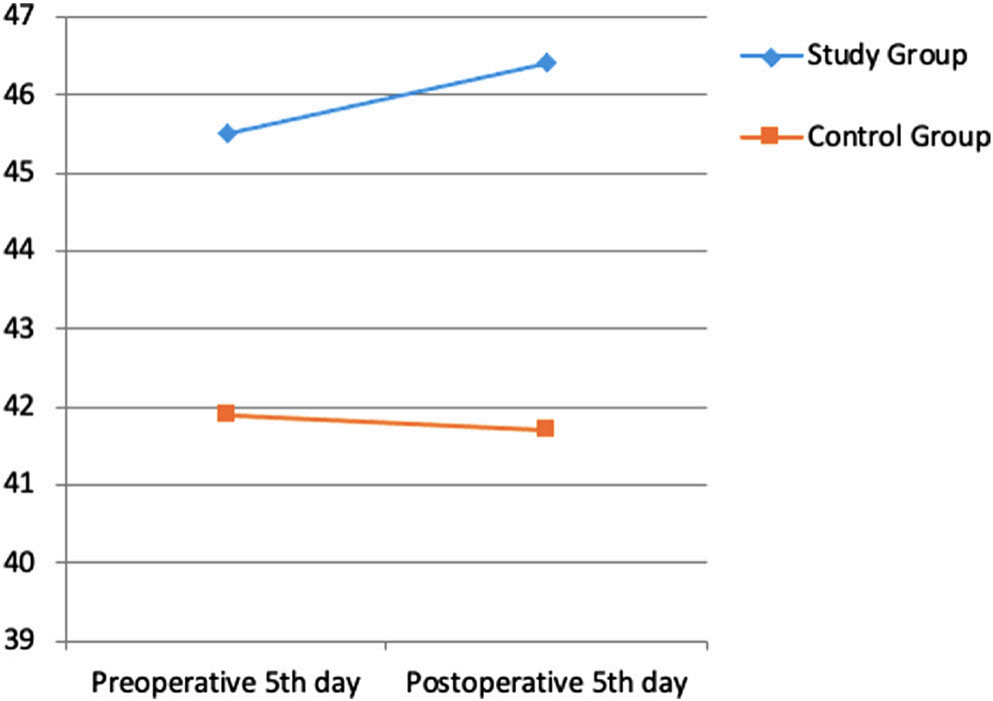
Difference in CD-4 values between pre-op and post-op.

**Figure 6.**
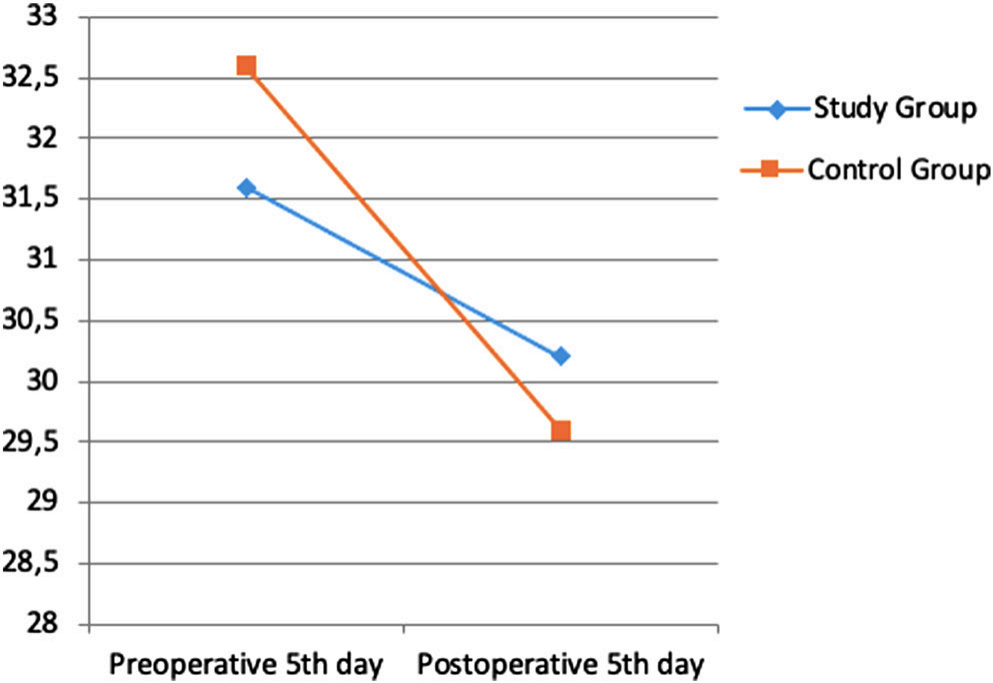
Difference in CD-8 values between pre-op and post-op.

**Figure 7.**
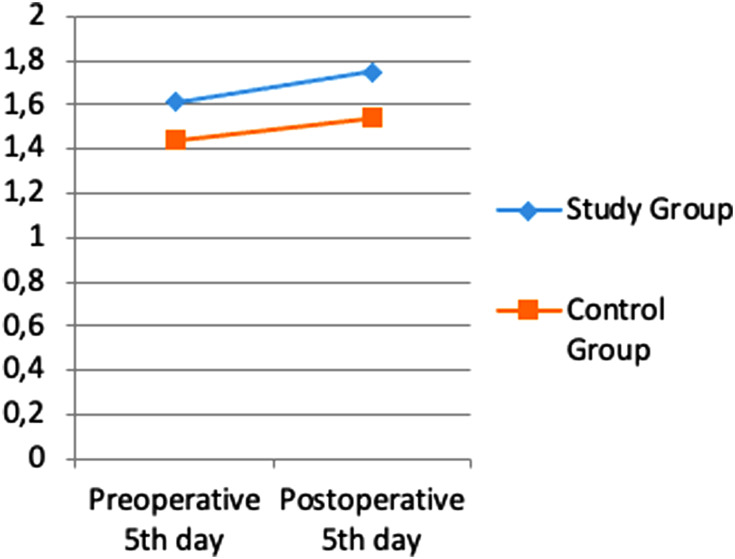
Difference in CD-4/CD-8 ratio between pre-op and post-op.

When CD-4 (Figure 5), CD-8 (Figure 6), and CD-4/CD-8 ratios (Figure 7), which are important indicators of cellular immunity, were examined, it was seen that there was no statistical difference in the preoperative period. However, it was observed that CD-4 level increased in the study group in the postoperative period, making a statistical difference, while it decreased minimally in the control group (*P* = .049). It was observed that there was no significant difference in CD-8 levels. There was an increase in the CD-4/CD-8 ratio in both groups, more prominently in the study group in the postoperative period, but there was no statistical difference in the preoperative and postoperative periods (*P* = .602/*P* = .680).

No statistically significant difference was found between the groups in the morbidity and mortality analysis. In terms of overall mortality, mortality developed in 5 (25%) patients in the study group and 4 (20%) patients in the control group in the following months (*P *= .705). In the general morbidity analysis, morbidity developed in 6 (30%) patients in the study group, while morbidity developed in 9 (45%) patients in the control group (*P* = .327). In the overall survival analysis performed with the Kaplan–Meier method, it was 21.8 ± 2.8 months in the study group and 19.1 ± 1.7 months in the control group (*P* = .859). The length of hospital stay was 12.89 ± 3.3 days in the patients in the study group, while it was 16.47 ± 6.83 days in the patients in the control group. The difference in the length of hospital stay was found to be statistically significant (*P* = .048) (Table 3).

Biliary fistula developed in 2 patients in the study group and 2 patients in the control group. While pancreatic fistula did not develop in the patients in the study group, pancreatic fistula developed in 2 patients in the control group (*P* = .147). In the postoperative follow-ups, DGE symptoms developed in 3 patients in the study group, while the same picture was observed in 9 patients in the control group (*P* = .038). In the analysis of postoperative wound infection findings, this picture was detected in 4 patients in the study group, while wound infection developed in 9 patients in the control group (*P* = .091) (Table 3).

When the histopathological reports of all patients were examined, it was seen that the tumor origin was the pancreatic head-uncus in 23 (57.5%) patients, the ampulla of Vater in 9 (22.5%), the second part of the duodenum in 5 (12.5%), and distal common bile duct and in 3 patients (7.5%). The histological type of the tumor was pancreatic adenocarcinoma in 20 patients (50%), ampullary adenocarcinoma in 8 patients (20%), duodenal adenocarcinoma in 5 patients (12.5%), biliary adenocarcinoma in 2 patients (5%), pancreatitis in 2 patients (5%), pancreatic neuroendocrine tumor (pNET) in 1 patient (5%), and chronic fibrosis in 1 patient (2.5%) (Table 4).

## Discussion

Periampullary region tumors include tumors arising 2 cm around the papilla Vater where the ampulla of Vater, which is formed by the junction of the main pancreatic duct and the common bile duct, opens into the duodenum. In order of decreasing incidence, these tumors are pancreatic head, ampulla of Vater, common bile duct, and duodenal tumors.^1^ Pancreatic ductal adenocarcinoma is the most common subgroup of tumors originating from the periampullary region, with 60%-70%. Pancreatic adenocarcinomas are typically characterized by aggressive growth and early systemic spread. It causes clinical symptoms in the early stages of the disease due to its close anatomical relationship with the ampulla of Vater. Therefore, it has better results than other tumors of the pancreas in terms of treatment options and prognosis.^9^ Although tumors of this region have different biological behavior and prognosis, they often show similar clinical features. The primary origin of the tumor cannot be identified with preoperative imaging methods. Pancreaticoduodenectomy is currently the only curative treatment method for tumors of the periampullary region, regardless of the primary tumor.^2^

This major surgical procedure, which includes multistage resection and reconstruction steps, stimulates the systemic inflammatory response. Systemic inflammatory response syndrome is a clinical phenomenon characterized by increased secretion of proinflammatory cytokines. Hypermetabolism secondary to uncontrolled systemic response may cause immune dysfunction, complications (SIRS, sepsis), and organ dysfunction after surgery. Adding cachexia and malnutrition caused by cancer biology to the existing inflammatory picture will result in the deterioration of the patient’s clinical condition.^10^ Determining a comprehensive nutritional assessment strategy in the preoperative period in patients planned for PD will reduce the morbidities that may develop.^11^

One of the main strategies recommended to reduce complications is to support surgical patients with EIN containing arginine, omega-3 fatty acids, and RNA (dietary nucleotides) both pre and postoperatively.^12^ It has been shown that the proposed immunonutrition optimizes the immune response and wound healing by providing essential nutrients necessary for the functioning of other immune agents, especially T lymphocyte activity.^8^

There are different studies on the timing of the administration of immunonutrition in patients with gastrointestinal cancer. When these studies are examined, it is seen that there are 3 different approaches. These are the approaches to be applied only in the preoperative period, to be applied in the perioperative (both preoperative and postoperative) period, and to be applied only in the postoperative period. In a study published by Giger et al, it was emphasized that the use of immunonutrition for only 3 days showed a decrease in inflammatory responses, but did not provide a significant clinical benefit.^10^ In another study by Braga et al, it was suggested that immunonutrition should be started in the preoperative period and continued for 5-7 days after surgery.^12^ In a randomized controlled study conducted by Gnotti et al in patients with gastrointestinal cancer whose nutritional level was standardized, it was reported that immunonutrition administered for 5 days preoperatively was as effective as perioperative treatment in reducing postoperative morbidity.^8^ By examining all these clinical data, we believe that nutritional support should be applied for at least 5 days in order to strengthen immunity and show maximum benefit. Therefore, in our study, we only applied enteral immunonutrition support for 5 days before surgery.

In a study by Silvestri et al^13^ in which the effect of preoperative immunonutrition was investigated in patients who underwent PD, hemoglobin, albumin, and total protein levels were evaluated under the heading of nutritional laboratory parameters, and no significant difference was found between the groups. In another study investigating the effect of preoperative immunonutrition in patients who underwent PD by Aida et al,^14^ it was observed that there was no significant difference in favor of the immunonutrition group in albumin, transferrin, and total protein levels. In our study, albumin, transferrin, and total protein levels were examined as nutritional status indicators. Considering the pre- and postoperative periods in albumin levels, similar decrease rates were found in both groups. A more limited decrease was observed in transferrin and total protein levels in the study group. However, these changes were not found to be statistically significant.

In a study by Hamza et al^15^ investigating the effect of preoperative immunonutrition on mucosal immunity and inflammatory response in patients who underwent PD, levels of CD-4, CD-8, and CD-4/CD-8 ratio in 37 patients divided into 2 groups was examined in the preoperative and postoperative period. It was found that the ratio of CD-4 and CD-4/CD-8 increased significantly in the study group between the third and seventh postoperative days. Similarly, in our study, CD-4, CD-8, and CD-4/CD-8 ratio, which were measured twice, on the fifth day before the operation and the fifth day after the operation, were compared. It was observed that there was no statistical difference in the preoperative period, but the CD-4 levels in the postoperative period increased significantly in the study group, while it decreased minimally in the control group. It was observed that there was no significant difference in CD-8 levels. There was an increase in the CD-4/CD-8 ratio in both groups in the postoperative period, more prominently in the study group. It was not statistically significant.

Immunoglobulin alpha (IgA) is one of the most produced antibodies in the blood.^16^ The majority of IgA is secreted from the intestinal lumen as an effector protein of mucosal-associated lymphoid tissue and helps to protect the intestinal mucosa from microorganisms.^17^ When IgA levels, which is one of the most important immune markers in terms of mucosal defense functions, were compared in our study, it was observed that while the mean IgA level was preserved in the study group in the pre- and postoperative period, it was decreased in the control group. No statistically significant difference was found.

In a meta-analysis of 21 randomized controlled trials and 2730 patients published by Cerantola et al, it was shown that preoperative immunonutrition reduces morbidity and hospital stay, but does not affect mortality in patients undergoing gastrointestinal surgery.^18^ Two similar studies were conducted by Marik and Zaloga^19^ and Bozzetti et al,^20^ which emphasized that perioperative immunonutrition is a protective independent factor in reducing infectious complications in a multivariate model in patients undergoing major abdominal surgery. In a study by Shirakawa et al,^21^ in which the effect of preoperative immunonutrition was investigated in patients who underwent PD with 31 patients, it was suggested that immunonutrition was effective in preventing wound infection and reducing the surgical stress response. In the same study, similar results were found in both groups in terms of mortality, length of hospital stay, DGE, pancreatic fistula, and other morbidities.^21^

In another study by Aida et al^14^ with 50 patients, it was emphasized that preoperative immunonutrition in patients who underwent PD reduced postoperative complications by modulating PGE2 production and T cell differentiation. In the same study, complications developed in total infectious complications (wound infection, intra-abdominal abscess, pneumonia, sepsis) in 7 patients (28%) in the EIN group and 15 patients (60%) in the control group, which was statistically significant (*P* < .05). It was stated that there was no significant difference between the 2 groups in terms of noninfectious complications (DGE, pancreatic fistula, and intra-abdominal bleeding).

One of the most recent studies on this subject is the study by Silvestri et al,^13^ in which the effect of preoperative immunonutrition was investigated in patients who underwent PD. As a result of this study conducted by Silvestri et al^13^ with 96 patients, it was found that there was a difference between the groups in terms of mortality (2.1% in each group) and overall morbidity rate (41.6% vs. 47.9%/*P* > .05) in terms of complications related to pancreatic surgery. No statistical difference was found. In contrast, in the immunonutrition group, infectious complications (22.9% vs. 43.7%/*P* < .05) and length of hospital stay (18.3 ± 6.8 days vs. 21.7 ± 8.3 days/*P* < .05), a statistically significant decrease was reported. In our study, similar to the literature, no significant difference was found between the groups in terms of mortality and morbidity (*P* > .05). No statistically significant difference was found in the overall survival analysis between the groups, either (21.8 ± 2.8 months vs. 19.1 ± 1.7 months/*P *> .05). Wound infection developed in 4 patients in the immunonutrition group and 9 patients in the control group. Although this result is not statistically significant (*P *= .091), we think it is remarkable. It was observed that DGE developed less frequently in the study group compared to the control group, and the duration of hospital stay was shorter in the study group. Both findings were statistically significant (*P* < .05).

Due to the coronavirus disease 2019 pandemic, the number of patients enrolled in our study fell short of the originally planned sample size. This limitation represents a significant constraint to our study, as it may have affected the power of our statistical analyses. A larger sample size is required to improve the generalizability of our findings. Therefore, further studies with larger sample sizes are warranted to confirm our results.

The number of studies investigating the efficacy of enteral immunonutrition in patients undergoing PD is limited in the literature. The effects of these products, which are increasingly used in surgical patients, continue to be examined in clinical studies. The results of this prospective randomized controlled study conducted by us are in agreement with the literature in terms of general findings. Further clinical studies are needed in this regard.

## Figures and Tables

**Table 1.** Enteral Immunonutrition Formula Ingredients

Ingredients	Amount
Protein	18 (g/237 mL)
Carbohydrates	44.8 (g/237 mL)
Fat	9.2 (g/237 mL)
Fibers	3.3 (g/237 mL)
Arginine	4.3 (g/237 mL)
Omega-3 fatty acids	1.4 (g/237 mL)
RNA	0.43 (g/237 mL)
Na	150 (mg/100 mL)
Zn	2.1 (mg/100 mL)
Ca	114 (mg/100 mL)
P	101 (mg/100 mL)
K	190 (mg/100 mL)
Fe	1.7 (mg/100 mL)
Cl	169 (mg/100 mL)
Mg	32 (mg/100 mL)

**Table 2.** Demographics of the Patients

	Study Group	Control Group	Total	*P*
Number of patients	20	20	40	
Gender				
Male	12 (60%)	14 (70%)	26 (65%)	.507
Female	8 (40%)	6 (30%)	14 (35%)	
Age	60.95 ± 11.13	62.05 ± 9.99	61.50 ± 10.45	.744
BMI	26.42 ± 2.93	26.34 ± 2.69		.942
ASA score				
I	2	0	2	.290
II	15	18	33	
III	3	2	5	
NRS score				
I	4	1	5	.420
II	8	7	15	
III	8	12	20	
Smoking	9 (45%)	12 (60%)	21 (5.,5%)	
Alcohol	5 (25%)	5 (25%)	10 (25%)	
Comorbid diseases				
I	8 (40%)	9 (45%)	17 (42.5%)	
II	6 (30%)	5 (25%)	11 (27.5%)	
CEA	3.71 ± 5.46	4.64 ± 4.07	4.18 ± 4.78	.545
Ca 19-9	156.50 ± 508.95	254.99 ± 435.10	205.790 ± 470.01	.515
Blood type				
A	7	9	16 (40%)	
B	1	4	5 (12,5%)	
O	12	6	18 (45%)	
AB	0	1	1 (2.5%)	
Rh				
Rh-	1	2	3 (7.5%)	
Rh+	19	18	37 (92.5%)	

**Table 3.** Mortality and Morbidity Data of the Patients

	Study Group	Control Group	Total	*P*
Overall survival (median, months)	21.8 ± 2.8 (95% CI)	19.1±1.7 (95% CI)	22.5±1.9 (95% CI)	.859
LOS (days)	12.89±3.3	16.47±6.83	14.68±5.61	.048
Mortality	5 (25%)	4 (20%)	9 (22.5%)	.705
Morbidity	6 (30%)	9 (45%)	15 (37.5%)	.327
Biliary fistula	2 (10%)	2 (10%)	4 (10%)	1
Pancreatic fistula	0	2 (10%)	2 (5%)	.147
DGE	3 (15%)	9 (45%)	12 (30%)	.038
Surgical site infection	4 (20%)	9 (45%)	13 (32.5%)	.091

DGE, delayed gastric empty; LOS, length of hospital stay.

**Table 4.** Histopathological Data

	Study Group	Control Group	Total
Origin of tumor			
				Pancreas head-uncus	10	13	23 (57.5%)
				Ampulla of Vater	6	3	9 (22.5%)
				Second part of duodenum	3	2	5 (12.5%)
Distal common bile duct	1	2	3 (7.5%)
Type of tumor			
				Pancreatic adenocarcinoma	9	11	20 (50%)
				Ampullary adenocarcinoma	5	3	8 (20%)
				Duodenal adenocarcinoma	3	2	5 (12.5%)
				Biliary adenocarcinoma	0	2	2 (5%)
				Pancreatitis	1	1	2 (5%)
				pNET	1	1	2 (5%)
Chronic fibrosis	1	0	1 (2.5%)

pNET, pancreatic neuroendocrine tumor.
